# Multi-strain synbiotic and lifestyle modifications on patients with metabolic dysfunction-associated steatotic liver disease (MASLD): a randomized double-blinded placebo-controlled trial

**DOI:** 10.1186/s40001-026-03901-3

**Published:** 2026-01-30

**Authors:** Masoud Faghieh Dinavari, Samaneh Abbasian, Amirreza Jabbaripour Sarmadian, Tahereh Vaezi, Tayyebeh Vaezi, Zeinab Nikniaz, Ali Riazi

**Affiliations:** Liver and Gastrointestinal Diseases Research Center, Tabriz University of Medical Sciences, Tabriz, Iran

**Keywords:** Nonalcoholic fatty liver disease, NAFLD, Probiotic, Synbiotic, Lifestyle, Randomized controlled trial

## Abstract

**Background:**

MASLD is the most common chronic liver disease and the leading cause of liver-related morbidity and mortality globally. While probiotic supplementations show promise as adjunctive therapy, existing trials are limited by the quality of evidence, small sample sizes, and methodological inconsistencies, highlighting the need for well-designed studies, particularly on multi-strain synbiotics. Therefore, we conducted this trial to evaluate the effects of multi-strain synbiotic combined with lifestyle modifications, including an individualized diet and physical activity program, on patients with MASLD.

**Methods:**

In this randomized, double-blinded, placebo-controlled trial, 80 MASLD patients were enrolled and underwent lifestyle modifications. Participants were randomized in a 1:1 ratio to receive either synbiotic capsules (500 mg each, 10^9^ CFU per capsule) containing two strains of *Bifidobacteria*, two strains of *Lacticaseibacillus*, two strains of *Lactobacillus*, and one strain of *Streptococcus*, as well as Fructooligosaccharides as prebiotic, or placebo (one capsule every 12 h) for 12 weeks. All patients were evaluated both at the beginning and the end of the trial in terms of anthropometric parameters (weight, BMI, and WC), hematological parameters (Hb and Plt), coagulation status (PT and INR), lipid profile (TG, TC, HDL-C, and LDL-C), FBS, liver function (ALT and AST), and liver inflammatory biomarkers (TNF-α and IL-6).

**Results:**

No significant changes were observed in anthropometric measures either within or between groups during the study period. Moreover, analysis of venous blood samples at the end of the trial showed significant improvements in some measures of lipid profile (TC and LDL-C), FBS, liver function (ALT and AST), and liver inflammatory biomarkers (TNF-α and IL-6) in the synbiotics-receiving group compared to both the beginning of the trial and the placebo-receiving group. However, no significant changes were observed in hematological parameters (Hb and Plt), coagulation status (PT and INR), and lipid profile (TG and HDL-C) compared to the beginning of the trial and the placebo-receiving group. Supplements were tolerated well, with no complications or allergic reactions reported.

**Conclusions:**

Supplementation with multi-strain synbiotics, in combination with lifestyle modifications, could serve as a promising adjunctive therapy to control disease progression in patients with MASLD.

## Introduction

Metabolic dysfunction-associated steatotic liver disease (MASLD), previously known as nonalcoholic fatty liver disease (NAFLD), is the most common chronic liver condition around the world, affecting approximately 38% of adults and 7–14% of children and adolescents [1]. It is characterized by the accumulation of triglyceride (TG) and free fatty acid in hepatocytes and is diagnosed in the presence of at least one of the five cardiometabolic risk factors, as outlined by the Delphi consensus statement [2, 3]. MASLD has the potential to progress into severe liver conditions, including liver fibrosis, cirrhosis, and hepatocellular carcinoma (HCC), making it the leading cause of liver-related morbidity and mortality globally [1, 4]. Consequently, improved management strategies are urgently needed to address this growing public health challenge.

Despite the various pharmacological and non-pharmacological treatments proposed for this disease, lifestyle modifications, particularly changes in dietary habits and physical activity, remain the cornerstone of MASLD management [5–7]. Given the direct blood interactions between the intestine and liver, as well as the emerging role of gut microbiota on disease progression [8, 9], probiotics have been proposed as a potential adjunctive therapy to attenuate disease advancement [10].

In line with this, the consumption of probiotics and prebiotics has been reported to be associated with a reduced risk of MASLD in adults, further supporting their potential role in early management strategies [11]. Moreover, several clinical trials have evaluated the effects of these supplements in patients with NAFLD, generally reporting promising results across a range of outcomes, including anthropometric measures, lipid profile, glycemic control, liver function, liver inflammatory biomarkers, and hepatic steatosis [12–17].

However, meta-analyses have reported varying degrees of efficacy across different metabolic and hepatic parameters [18–20], with GRADE methodology assessments revealing predominantly low- and very low-quality evidence for synbiotics' effects on NAFLD-related outcomes [20]. These quality limitations may be attributed to trials being constrained by small sample sizes, heterogeneous study designs, and substantial variations in supplement strains, dosages, and treatment durations, which collectively complicate result interpretation and impede the development of standardized therapeutic guidelines. Moreover, trials specifically evaluating multi-strain synbiotics formulations remain limited, highlighting a knowledge gap, that should be prioritized in future research endeavors [14–16].

Considering all these evidence quality limitations and methodological constraints, we conducted a randomized, double-blinded, placebo-controlled trial to evaluate the effects of seven-strain synbiotics combined with lifestyle modifications, including an individualized diet and physical activity program, on anthropometric measures, hematological parameters, coagulation status, lipid profile, fasting glycemic control, liver function, and liver inflammatory biomarkers in patients with MASLD.

## Methods

### Study design

This study was designed and conducted as a randomized, double-blinded, placebo-controlled trial between March and June 2022 at the gastroenterology clinic of Imam Reza Hospital in Tabriz City. The trial followed the consolidated standards of reporting trials (CONSORT) 2010 guidelines to ensure methodological transparency and reporting quality. All eligible MASLD patients initially underwent controlled lifestyle modifications as their only approved treatment, including an individualized diet and physical activity program, and were then randomized in a 1:1 ratio to receive either capsules of synbiotic or placebo (one capsule every 12 h) for 12 weeks (3 months). Anthropometric measures, hematological parameters, coagulation status, lipid profile, fasting glycemic control, liver function, and liver inflammatory biomarkers were assessed both at baseline and end of the trial.

### Sample size

The sample size was calculated using PASS software based on interleukin (IL)−6 reduction data from Loguercio et al. [21], with a statistical power of 80%, a 95% confidence level, and an anticipated 10% dropout rate. The minimum required sample size was 40 participants per group (synbiotics and placebo), totaling 80 participants.

### Inclusion and exclusion criteria

We enrolled patients aged 18–75 years, without any gender limitations, who were diagnosed with MASLD based on mild-to-moderate hepatic steatosis confirmed by FibroScan, along with at least one of the five key cardiometabolic risk factors, including overall or central obesity, impaired glucose regulation or type 2 diabetes mellitus (T2DM), high blood pressure, elevated TG levels, or reduced high-density lipoprotein cholesterol (HDL-C) levels [2].

Patients were excluded if they had excessive alcohol consumption (> 20 g/day for females and > 30 g/day for males), liver cirrhosis, HCC, recent antibiotic use (within the past 3 months), use of hepatotoxic medications, current pregnancy or breastfeeding, thyroid disorders, or regular use of probiotics, prebiotics, or synbiotics as dietary supplements or components of their daily diet. Furthermore, patients were excluded if they developed allergies or adverse reactions to the supplements, required antibiotics due to medical conditions, consumed probiotic-containing products during the trial, or showed poor adherence to the trial protocol.

### Eligible patients

At the beginning of the trial, all volunteer participants were thoroughly evaluated by two internists according to the inclusion and exclusion criteria. Eligible participants were then provided with a comprehensive explanation of the study’s objectives, interventions, procedures, assessments, and potential risks, along with the opportunity to ask any questions. Then, they were informed about the two types of supplements (synbiotics or placebo) and explicitly told that they would not be aware of which one they were receiving. They were also assured that participation was entirely voluntary, that they could withdraw from the study at any time without any impact on their routine medical care, and that all personal information would be kept strictly confidential. Those who agreed to participate provided written informed consent before enrollment.

### Lifestyle modifications

Following enrollment, we assessed both the dietary intake and physical activity of participants using their 1-week diet and activity records, respectively. Then, daily calorie intake was calculated using Nutritionist IV software based on these records. A trained nutritionist provided each patient with an individualized weight-loss diet, designed to be 500 cal below their estimated daily intake. The diet comprised 50–60% carbohydrates, 25–35% protein, and 5–15% fat, tailored to each patient’s profile and socioeconomic status. In addition, all patients were advised to avoid foods containing probiotics and prebiotics, and suitable alternatives were incorporated into their dietary programs.

To complement the lifestyle modifications, an individualized physical activity program was designed for each participant based on their 1-week activity records. This program included simple exercises, such as moderate-paced walking (slow jogging), stretching, and playing with a ball outdoors, to be performed three times a week for 30–60 min per session.

### Randomization process

The blocked randomization method was employed to allocate participants into intervention (synbiotics) or control (placebo) groups, ensuring balanced group sizes. Block sizes and the total number of participants were predefined, and within each block, random sequences were generated to guarantee equal distribution between the two groups. To preserve allocation concealment and minimize selection bias, sealed, opaque envelopes containing the randomization codes were prepared. Each participant was assigned to a group based on the code within their respective envelope. This process was carried out by an independent third party who was not involved in patient recruitment, assessment, or data analysis.

### Interventions

In this trial, we administered *Familact®* capsules, which are multi-strain (seven-strain) synbiotics (500 mg each, 10^9^ CFU per capsule) containing two strains of *Bifidobacteria* (*Bifidobacterium breve* and *Bifidobacterium longum*), two strains of *Lacticaseibacillus* (*Lacticaseibacillus rhamnosus* and *Lacticaseibacillus casei*), two strains of *Lactobacillus* (*Lactobacillus bulgaricus* and *Lactobacillus acidophilus*), one strain of *Streptococcus* (*Streptococcus thermophilus*), and Fructooligosaccharides (FOS) as prebiotics. Identical-appearing placebo capsules were produced to match the synbiotics capsules in shape, color, taste, smell, and packaging. Both synbiotics and placebo capsules were manufactured by Zist Takhmir® Pharmaceutical Company. We delivered supplements to each patient every 2 weeks at the gastroenterology clinic and evaluated whether they had adhered to the trial protocol using supplements and following the diet and physical activity program. The supplements were delivered by an independent third party who was not involved in patient recruitment, assessment, or data analysis.

### Assessments

At the beginning of the trial, demographic data and medical history were collected through interviews conducted by two internists. Patients were examined both at the baseline and the end of the trial for anthropometric parameters, including weight, height, body mass index (BMI), and waist circumference (WC). In addition, 5cc of venous blood was collected after an 8-h fasting period from each patient both at the baseline and end of the trial. All blood samples were processed in the same laboratory setting at Imam Reza Hospital in Tabriz City. The blood samples were analyzed for various parameters, including hematological parameters (hemoglobin (Hb) and platelet (Plt)), coagulation status [prothrombin time (PT) and international normalized ratio (INR)], lipid profile (TG, total cholesterol (TC), HDL-C, and low-density lipoprotein cholesterol (LDL-C)), fasting blood sugar (FBS), liver function (alanine aminotransferase (ALT) and aspartate aminotransferase (AST)), and liver inflammatory biomarkers [tumor necrosis factor-alpha (TNF-α) and IL-6]. Since this study was conducted as a double-blinded trial, all researchers assessing the patients were unaware of which supplement each patient received.

### Follow-up and safety considerations

To monitor adherence to the study protocol, participants were provided with a tracking sheet to record daily use of the supplement as well as compliance with the prescribed diet and physical activity program. Given the importance of lifestyle modifications in this study, both participants and their family members were strongly encouraged to complete the sheet honestly and, when possible, to support adherence through home monitoring. The sheets were reviewed biweekly, and based on the reported adherence, supplements for the following 2 weeks were dispensed.

Moreover, patients were monitored for any potential complications or allergies during the trial and for 1 month following its completion. In case of any unforeseen events, patients were instructed to discontinue the use of supplements immediately and refer to the gastroenterology clinic or emergency department as soon as possible.

### Statistical analysis and data interpretation

Data were collected and analyzed using SPSS software version 25. The Kolmogorov–Smirnov test was applied to assess the normality of quantitative variables. Mean and standard deviation were calculated and reported for continuous variables, while frequencies and percentages were used for nominal and ordinal variables. For within-group comparisons, a paired sample *t* test was used, and for between-group comparisons, the independent sample *t* test was applied. The chi-squared test was employed to compare categorical variables; however, Fisher’s exact test was used wherever necessary. In all analyses, a *p* value of less than 0.05 was considered statistically significant.

### Ethics

The study protocol was approved by the ethics committee of Tabriz University of Medical Sciences with the approval ID IR.TBZMED.REC.1400.1095 on 19/01/2022. In addition, the trial was registered with the Iranian Registry of Clinical Trials (IRCT) under the registration number IRCT20220104053626N3 on 22/02/2022. The registration details are available at https://irct.behdasht.gov.ir/trial/62070.

## Results

**Fig. 1:**
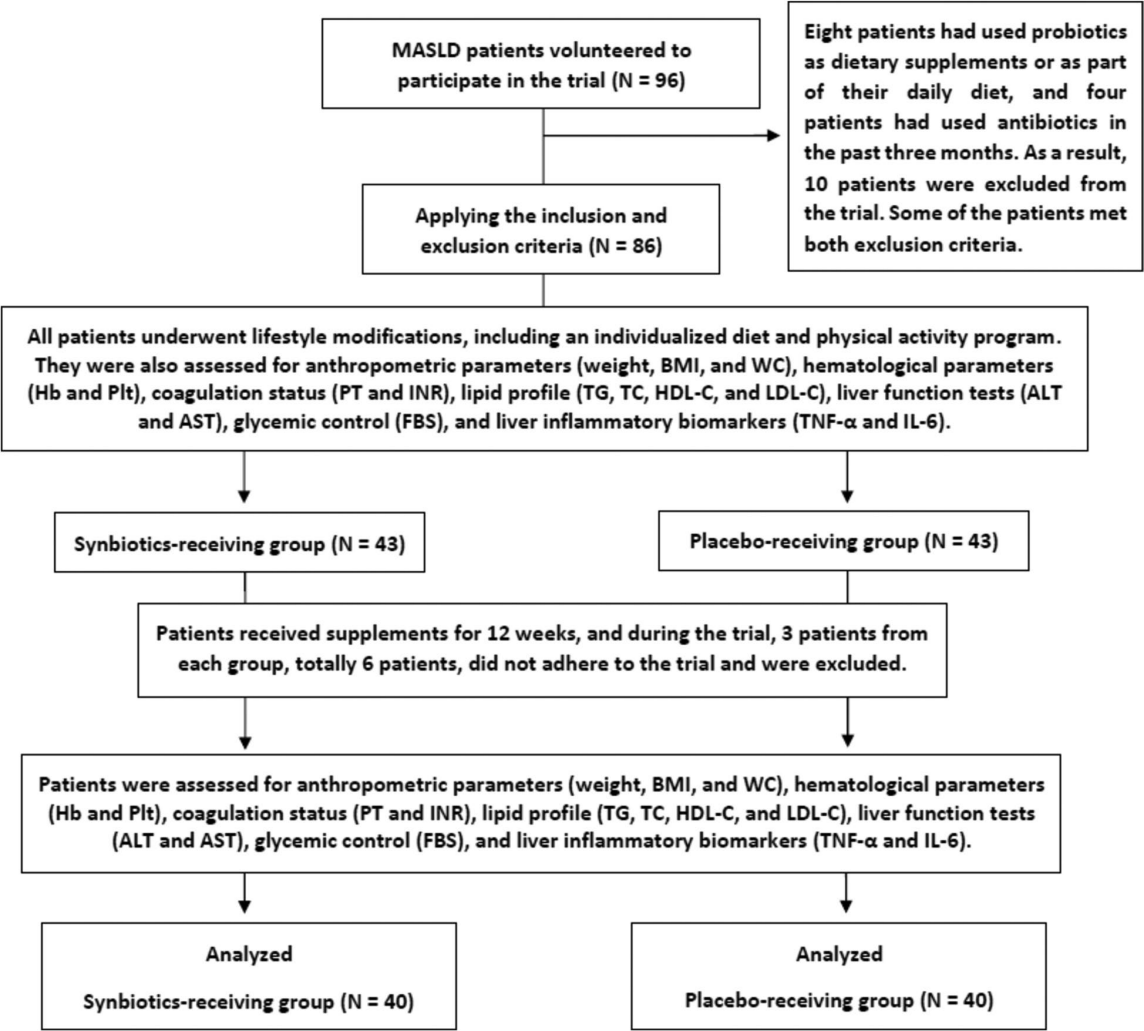
CONSORT flow diagram of the trial process

In this trial, 96 patients initially volunteered to participate. After applying the inclusion and exclusion criteria, 86 patients were enrolled. However, six patients were excluded during the study due to non-adherence to the trial protocol, resulting in a dropout rate of 7.0%. Ultimately, 80 patients with MASLD were included in the final analysis, with 40 participants in each group (synbiotics and placebo), all of whom underwent lifestyle modifications (Fig. 1). The collected data were normally distributed. Of the participants, 34 (42.5%) were male and 46 (57.5%) were female, with a mean age of 49.01 ± 12.19 years. At baseline, there were no statistically significant differences between the synbiotics and placebo receiving groups in terms of age, gender, and disease follow-up duration (*p* value > 0.05), as presented in Table 1.

**Table 1:** Baseline characteristics of the patients

Variables (Unit)		Synbiotics-receiving group	Placebo-receiving group	*p* value
Number of Patients		40	40	–
Age (years)		49.37 ± 12.36	48.14 ± 10.29	0.559^*^
Gender	Male (N)	18 (45%)	16 (40%)	0.882**
	Female (N)	22 (55%)	24 (60%)	
Disease Follow-up Duration (months)		12.96 ± 3.35	12.49 ± 3.96	0.854^*^

*Independent sample *t* test ** Fisher’s exact test

All baseline and post-intervention data for the measured parameters have been fully reported for all 80 patients without any omissions. At the beginning of the trial, there were no statistically significant differences between the synbiotics and placebo receiving groups in terms of anthropometric parameters (weight, height, BMI, and WC), hematological parameters (Hb and Plt), coagulation status (PT and INR), lipid profile (TG, TC, HDL-C, and LDL-C), FBS, liver function (ALT and AST), and liver inflammatory biomarkers (TNF-α and IL-6) (*p* value > 0.05), as shown in Tables 2 and 3.

**Table 2:** Anthropometric parameters of the patients at the beginning and end of the trial

Variables (Unit)		Synbiotics-receiving group	Placebo-receiving group	*p* value
Height (Cm)	Baseline	170.03 ± 23.10	174.23 ± 25.96	0.509^*^
	After Intervention	170.03 ± 23.10	174.23 ± 25.96	0.509^*^
	*p* value	0.999**	0.999**	–
Weight (Kg)	Baseline	94.01 ± 4.15	92.89 ± 5.34	0.480^*^
	After Intervention	92.42 ± 2.89	90.22 ± 5.59	0.411^*^
	*p* value	0.356**	0.236**	–
BMI	Baseline	31.11 ± 5.03	30.01 ± 5.97	0.613^*^
	After Intervention	28.24 ± 3.19	28.14 ± 5.19	0.569^*^
	*p* value	0.356**	0.289**	–
WC (Cm)	Baseline	95.40 ± 4.85	92.14 ± 4.32	0.419^*^
	After Intervention	93.55 ± 3.27	91.86 ± 4.95	0.259^*^
	*p* value	0.189**	0.431**	–

*Independent sample *t* test ** A paired samples *t* test

**Table 3:** Analysis of venous blood samples from patients at the beginning and end of the trial

Variables (Unit)			Synbiotics-receiving group	Placebo-receiving group	*p* value
Hematological Parameters	Hb (mg/dL)	Baseline	14.7 ± 9.11	14.5 ± 11.29	0.896^*^
		After Intervention	14.02 ± 6.24	14.85 ± 3.59	0.801^*^
		*p* value	0.603**	0.559**	–
	Plt (× 1000/mm^3^)	Baseline	425.95 ± 100.20	411.59 ± 95.59	0.359^*^
		After Intervention	412.59 ± 89.59	401.89 ± 85.49	0.411^*^
		*p* value	0.662**	0.554**	–
Coagulation Status	PT (Sec.)	Baseline	11.59 ± 2.24	12.48 ± 2.55	0.589^*^
		After Intervention	11.01 ± 2.85	12.08 ± 2.14	0.803^*^
		*p* value	0.745**	0.789**	–
	INR (Index)	Baseline	1.0 ± 0.20	1.0 ± 0.15	0.555^*^
		After Intervention	1.0 ± 0.14	1.0 ± 0.18	0.741^*^
		*p* value	0.801**	0.788**	–
Lipid Profile	TG (mg/dL)	Baseline	181.19 ± 25.25	175.58 ± 21.59	0.442^*^
		After Intervention	171.24 ± 20.15	170.96 ± 21.44	0.245^*^
		P-value	0.425**	0.581**	-
	TC (mg/dL)	Baseline	195.15 ± 24.26	200.25 ± 25.63	0.663^*^
		After Intervention	183.85 ± 24.41	194.45 ± 24.8	0.033^*^
		*p* value	0.001**	0.569**	–
	LDL-C (mg/dL)	Baseline	118.89 ± 20.96	125.85 ± 25.63	0.259^*^
		After Intervention	104.33 ± 15.24	120.25 ± 20.96	0.024^*^
		*p* value	0.011**	0.254**	–
	HDL-C (mg/dL)	Baseline	47.85 ± 5.59	44.89 ± 5.59	0.659^*^
		After Intervention	48.24 ± 5.96	45.19 ± 5.85	0.711^*^
		*p* value	0.854**	0.759**	–
Glycemic Control	FBS (mg/dL)	Baseline	127.84 ± 6.55	125.46 ± 6.98	0.786^*^
		After Intervention	116.23 ± 5.87	121.51 ± 6.23	0.001^*^
		*p* value	0.001**	0.474**	–
Liver Function	ALT (IU/L)	Baseline	57.10 ± 5.22	58.25 ± 5.32	0.701^*^
		After Intervention	42.24 ± 3.14	55.48 ± 5.59	0.001^*^
		*p* value	0.001**	0.475**	–
	AST (IU/L)	Baseline	47.10 ± 7.96	44.25 ± 6.15	0.558^*^
		After Intervention	34.14 ± 5.29	41.87 ± 5.59	0.039^*^
		*p* value	0.009**	0.631**	–
Liver Inflammatory Biomarkers	TNF-α (pg/ml)	Baseline	3.66 ± 1.29	3.59 ± 1.19	0.558^*^
		After Intervention	2.48 ± 1.85	3.55 ± 1.24	0.025^*^
		*p* value	0.014**	0.624**	–
	IL-6 (pg/ml)	Baseline	17.65 ± 2.28	18.10 ± 2.29	0.259^*^
		After Intervention	13.96 ± 2.85	17.59 ± 2.18	0.005*
		*p* value	0.032**	0.593**	–

*Independent sample *t* test **A paired samples *t* test

Variables with bolded and underlined values indicate statistically significant differences (*p* value < 0.05)

At the end of the trial, anthropometric parameters, including weight, BMI, and WC, were assessed. No significant changes were observed in these measures either within or between groups during the study period (*p* value > 0.05), as shown in Table 2. Moreover, analysis of venous blood samples at the end of the trial showed significant improvements in some measures of lipid profile (TC and LDL-C), FBS, liver function (ALT and AST), and liver inflammatory biomarkers (TNF-α and IL-6) in the synbiotics-receiving group compared to both the beginning of the trial and the placebo-receiving group, as shown in Table 3. However, no significant changes were observed in hematological parameters (Hb and Plt), coagulation status (PT and INR), and some measures of lipid profile (TG and HDL-C) in the synbiotics-receiving group compared to the beginning of the trial and the placebo-receiving group (*p* value > 0.05), as shown in Table 3.

All patients tolerated synbiotics and placebo capsules well; none reported any complications or allergic reactions, even at mild intensities.

## Discussion

The results of our trial showed that supplementation with multi-strain synbiotics combined with lifestyle modifications, including an individualized diet and physical activity program, for 12 weeks significantly improved some measures of lipid profile (TC and LDL-C), FBS, liver function (ALT and AST), and liver inflammatory biomarkers (TNF-α and IL-6) in MASLD patients. To the best of our knowledge, this is the first clinical trial conducted on patients diagnosed with MASLD based on the new definition, whereas previous studies were primarily conducted on patients meeting the earlier NAFLD criteria.

The term MASLD was introduced in 2023, replacing NAFLD, through a Delphi consensus statement led by major liver associations and developed by 53 global experts. This revision provides a more precise and inclusive classification that better reflects the metabolic basis of the disease while minimizing potential misinterpretations [2, 3]. This new definition expands the diagnostic criteria to include the coexistence of other liver diseases, such as chronic viral hepatitis, introduces a threshold for limited alcohol consumption, and enhances applicability to lean individuals with liver steatosis. However, over 95% of individuals previously diagnosed with NAFLD still meet the diagnostic criteria for MASLD [22–25]. Similarly, all patients in the present study fulfilled both sets of diagnostic criteria.

None of the pharmacological or non-pharmacological treatments have been able to meet the needs of all patients worldwide [5]. For example, Resmetirom, the only medication approved by the U.S. Food and Drug Administration (FDA) for patients with stage 2–3 fibrosis, marks a significant advancement in treatment. However, it is not available or beneficial for all MASLD patients globally [26]. Therefore, lifestyle modifications, including healthy dietary habits and increased physical activity, serve as the cornerstone of managing this condition [7]. Since we aimed not to interfere with patients’ routine medical care, we applied these lifestyle changes to all participants, regardless of whether they were in the intervention or control group.

Moreover, modifying the composition of gut microbiota is an accessible treatment approach that offers significant potential benefits by influencing nutrient metabolism, restoring balance in bacterial metabolite signaling and dysbiosis, enhancing intestinal barrier function, decreasing intestinal permeability, and subsequently regulating the gut–liver axis through direct blood interactions between the intestine and the liver. These mechanisms may improve hepatic inflammation, insulin sensitivity, and lipid accumulation, ultimately controlling the disease progression, fibrotic cascades, and associated complications [27–29]. Probiotics, which are live microbial cultures or fermented dairy products containing beneficial bacteria, may act through interactions with the gut microbiota and host metabolic pathways. The effects of probiotics can be enhanced by prebiotics, which are non-digestible dietary components that selectively promote the growth, activity, or composition of probiotics or the host microbiota. The combination of probiotics and prebiotics is referred to as synbiotics, which may exert greater health benefits than either component alone [30].

Although physicians, nurses, and pharmacists are generally knowledgeable about these supplements, they made limited recommendations regarding probiotics [31, 32]. However, studies have shown their significant effects in various diseases [33, 34], including irritable bowel syndrome (IBS) [35, 36], inflammatory bowel disease (IBD) [37, 38], metabolic disorders [39], and even seemingly unrelated conditions, such as cardiovascular disease (CVD) [40, 41], complications during hospitalization [42, 43], and cancer [44].

In the present trial, we used a seven-strain synbiotic formulation, which was accessible to our research team and aligned with the mechanistic aims of the study. The formulation included two *Bifidobacterium* species, which are among the earliest microbial colonizers of the human gastrointestinal tract and remain an important component of the gut microbiota throughout life [45], being detectable in more than 90% of healthy adults, although their relative abundance typically does not exceed about 15% [46]. They play essential roles in maintaining intestinal homeostasis by strengthening mucosal integrity, producing short-chain fatty acids, participating in nutrients and bile acid metabolism, regulating immune responses, reducing inflammatory and oxidative processes, and suppressing pathogenic bacteria and harmful microbial metabolites, thereby contributing to improved metabolic regulation [47, 48]. It has been shown that *B. breve* and *B. longum* are associated with long-term gut microbiome stability in healthy individuals over 1 year [49]. Studies have also reported a reduced abundance of these species in patients with MASLD, indicating a disruption of these beneficial taxa in liver disease [50]. Moreover, a recently published meta-analysis by Chang et al. [51] reported that *Bifidobacterium*-based probiotic combinations may confer meaningful cardiometabolic benefits, particularly through improvements in lipid profiles, glucose metabolism, and inflammatory regulation. Notably, the two *Bifidobacterium* species included in our trial, *B. breve* and *B. longum*, are among the most frequently employed species in such formulations, further supporting the rationale for their selection [48, 50].

The synbiotic formulation used in this trial also included four *Lactobacillus* species, which have since been reclassified under the updated genera *Lacticaseibacillus* and *Lactobacillus*. These taxonomic revisions were formally published in 2020, when the genus *Lactobacillus* was reorganized into 25 genera based on whole-genome phylogenetic analyses. Specifically, *L. rhamnosus* and *L. casei* were reclassified into the genus *Lacticaseibacillus*, whereas *L. bulgaricus* and *L. acidophilus* retained their original genus, *Lactobacillus* [52]. These species gradually colonize the human gastrointestinal tract after birth and can persist as constituents of the gut microbiota throughout life [53]. Although their abundance is generally lower than that of Bifidobacterium, these species nevertheless make comparable contributions to intestinal homeostasis and overall metabolic regulation [54, 55]. It has been shown that *Lacticaseibacillus* and *Lactobacillus* species serve as important modulators of the gut–liver axis in chronic liver diseases. Emerging evidence further indicates that the abundance and composition of these taxa are disrupted in patients with MASLD compared with healthy individuals, reflecting a dysbiotic shift within the gut microbiota [56, 57]. Furthermore, experimental and clinical studies have shown that *Lactobacillus*-based probiotic supplementation can improve liver enzyme levels and ameliorate metabolic and inflammatory disturbances in MASLD, with species such as *L. rhamnosus*, *L. casei*, *L. bulgaricus*, and *L. acidophilus* being among those most frequently associated with these beneficial effects. These findings support the use of these species included in our formulation as promising adjuvant candidates for MASLD [56, 58].

The final strain included in our synbiotic formulation was *Streptococcus thermophilus*, a well-characterized lactic acid bacterium widely used in fermented dairy products and frequently incorporated into multi-strain probiotic formulations [59]. *S. thermophilus* contributes to gut health through its ability to produce lactic acid, enhance lactose digestion, and support the growth of other beneficial taxa, particularly *Bifidobacterium* and *Lactobacillus* [60]. Clinical investigations using multi-strain preparations that include *S. thermophilus* have reported improvements in lipid profiles, liver enzyme levels, and metabolic and inflammatory parameters in patients with MASLD, suggesting that this species may act synergistically with other probiotic strains to promote hepatometabolic benefits [58].

Obesity is a common comorbidity in MASLD patients and is closely associated with disease progression, as excessive adiposity promotes insulin resistance, chronic low-grade inflammation, and hepatic fat accumulation. These metabolic disturbances not only exacerbate liver steatosis and fibrosis but also increase the risk of cardiovascular complications and other obesity-related comorbidities [61, 62]. In this study, all participants had elevated BMI and were classified as having class 1 obesity according to standard BMI categories. Therefore, the findings should be interpreted with the understanding that they specifically reflect outcomes in this obese patient population.

In this study, synbiotic consumption had a significant effect on the lipid profiles, particularly by improving TC and LDL-C levels. In general, dyslipidemia is a clinically important condition frequently observed in individuals with obesity and is recognized as a modifiable risk factor for atherosclerosis and CVDs [63, 64]. Moreover, it appears to play a critical role in the progression and exacerbation of MASLD, and when coexisting with it, may intensify the risk and severity of cardiovascular complications [65]. Dyslipidemia is characterized by abnormal lipid profiles, including elevated TG (≥ 150 mg/dL), high TC (≥ 200 mg/dL), increased LDL-C (≥ 100 mg/dL), or reduced HDL-C (< 40 mg/dL in men and < 50 mg/dL in women). Although these thresholds are generally recommended, optimal lipid levels may vary among individuals depending on age, sex, and other cardiovascular risk factors [66]. Given that LDL-C is the primary target in dyslipidemia and lipid profile management [63], the observed significant reduction in LDL-C levels in this study highlights a clinically relevant outcome.

Furthermore, in this trial, synbiotic consumption was associated with a significant reduction in FBS levels. Both MASLD and T2DM share a bidirectional relationship, whereby each can exacerbate the other. This interaction ultimately accelerates the progression of MASLD, increasing the risk of advanced fibrosis, HCC, and cardiovascular complications [67]. Therefore, maintaining appropriate fasting glucose levels is critically important in managing MASLD. In this study, although baseline FBS values were only slightly above the commonly recommended cutoff for impaired fasting glucose (≥ 126 mg/dL), the post-intervention mean in the synbiotic group shifted to within the acceptable range. This observation suggests a potentially favorable effect of the synbiotic on fasting glucose levels.

Ultimately, synbiotic consumption resulted in a significant improvement in liver function, as indicated by reductions in ALT and AST levels, as well as in liver inflammatory biomarkers, including TNF-α and IL-6. Hepatocellular injury, such as that induced by MASLD, compromises the integrity of hepatocyte membranes, leading to the release of intracellular enzymes, most notably ALT and AST, into the bloodstream [68, 69]. ALT is more specific to hepatic tissue and typically exhibits greater elevations than AST in cases of hepatic steatosis. The AST/ALT ratio is widely applied in various clinical scoring systems to evaluate liver disease severity; however, this ratio can vary depending on the underlying etiology, as well as the severity and stage of liver damage [69–71]. Concurrently, Kupffer cells secrete pro-inflammatory cytokines, such as TNF-α and IL-6, which not only contribute to chronic systemic inflammation but also promote endothelial activation, leading to increased expression of vascular adhesion molecules and the progression of atherosclerosis and cardiovascular complications [72]. Our findings suggest that synbiotic intervention may be effective not only in mitigating hepatocellular injury but also in attenuating hepatic and systemic inflammation, highlighting its potential therapeutic role in the management of MASLD.

As mentioned, several clinical trials have evaluated the effects of these supplements in patients with NAFLD, generally reporting promising results. One of the earliest well-designed studies in this field was conducted by Eslamparast et al. [73] in Iran, where this study was also conducted. They enrolled 38 patients, fewer than in our trial, and used a synbiotic formulation with a similar strain composition but a lower microbial count per capsule (10⁹ vs. 2 × 10⁸ CFU). They assessed a broader range of anthropometric and glycemic parameters, as well as lipid profile, blood pressure, metabolic equivalent of task, and energy intake. They reported significant improvements in insulin resistance indices and in TG, TC, and HDL-C levels. However, their study did not assess several clinically important parameters that we included, such as liver function tests (ALT and AST) and inflammatory biomarkers (TNF-α and IL-6). Musazadeh et al. [16] conducted a systematic review and meta-analysis of trials using synbiotics on NAFLD patients from inception to June 2024; and included 18 clinical trials involving 1188 patients with NAFLD, with following outcomes:

1. Anthropometric parameters: They observed significant improvements in body weight and WC, though no significant changes were found in BMI, waist-to-height ratio (WHtR), or body fat percentage. In our trial, changes in body weight, BMI, and WC were not statistically significant. Notably, we did not evaluate WHtR or body fat percentage in this study.
2. Lipid profile: Significant improvements in TC levels were reported, while no significant changes were observed in TG, HDL-C, or LDL-C. In this study, we observed significant improvements in both TC and LDL-C, but no significant changes in TG or HDL-C.
3. Liver function: They reported significant improvements in ALT, AST, and gamma-glutamyl transpeptidase (GGT) levels, with no significant changes in alkaline phosphatase (ALP). Our findings were largely consistent, though we did not assess GGT or ALP in our trial.
4. Inflammatory biomarkers: Significant reductions in C-reactive protein (CRP) and TNF-α were observed. In this study, we also noted a significant improvement in TNF-α levels. However, we did not evaluate CRP; instead, we focused on IL-6, which decreased significantly.
5. Blood pressure: They reported a significant reduction in systolic blood pressure (SBP), although no significant changes were observed in diastolic blood pressure (DBP). These parameters were not assessed in this study.
6. Hepatic steatosis: They found no significant changes in hepatic fibrosis or hepatic steatosis. These parameters were not evaluated in our trial.

However, they did not assess parameters related to glycemic control in these patients. In a systematic review and meta-analysis by Mozaffari et al. [17], probiotic interventions in NAFLD patients were associated with significant improvements in glycemic control, as evidenced by reductions in HOMA–IR and insulin levels, while no significant changes were observed in FBS or hemoglobin A1c (HbA1c). In this study, we evaluated only FBS, which showed a significant improvement, decreasing from slightly above the cutoff to within the normal range after the intervention.

Maddineni et al. [20] assessed the quality of evidence regarding the effects of synbiotics on NAFLD-related outcomes using the GRADE methodology; however, the overall quality was found to be low. The review identified high-quality evidence only for the effect of synbiotics on FBS. Moderate-quality evidence supported their benefits on ALT, AST, WC, and insulin levels. In contrast, low-quality evidence was reported for improvements in TG, CRP, and TNF-α, while very low-quality evidence supported effects on liver stiffness, BMI, TC, HDL-C, LDL-C, HOMA-IR, and leptin. No evidence was reported for GGT, ALP, HbA1c, IL-6, and body fat mass. Although synbiotics appear promising for certain NAFLD-related parameters, the scarcity of high-quality evidence highlights the need for further well-designed clinical trials.

In addition to these parameters, we assessed the effects of synbiotics on hematological parameters and coagulation status in MASLD patients. Hematologic abnormalities, such as anemia, thrombocytopenia, and coagulopathy, are common in liver disease due to impaired hematopoiesis [74, 75]. Therefore, we assessed Hb, Plt, PT, and INR at baseline and the end of the trial to evaluate the potential hematologic effects of synbiotics supplementation. At baseline, all parameters were within normal ranges in all participants. Throughout the study, neither synbiotics nor placebo induced liver injury or caused significant changes in these hematologic markers.

Despite the overall positive findings, our trial had several limitations that should be considered in future studies. First, the relatively short duration of the study limits our ability to assess the sustainability and long-term effects of these supplements. Second, the modest sample size per group, which included participants from a specific population and ethnicity, may restrict the generalizability of the results to broader populations. In addition, this study employed a per-protocol analysis, excluding participants with poor adherence from the final analyses. While this approach strengthens the internal validity of the findings, it may reduce their applicability to real-world settings, where adherence to interventions can vary. In parallel, adherence to dietary and physical activity recommendations was self-reported, which could introduce reporting bias despite our efforts to encourage accurate and honest completion of tracking sheets by both participants and their family members. Furthermore, we did not assess certain parameters, such as hepatic steatosis, GGT, HOMA–IR, and insulin levels, which could provide further insights into the broader impact of synbiotics on liver health. In addition, assessing changes in the fecal microbiota from the beginning to the end of the study and evaluating their response to supplementation, analyses that were not performed in this trial, would be essential for a more comprehensive understanding of gut–liver axis modulation. Given these limitations, future studies with larger sample sizes and extended trial durations are warranted. Such studies should also include additional demographic variables, such as race and socioeconomic status, to enhance the assessment of external validity and generalizability. Moreover, evaluating a wider range of relevant parameters would allow for a more comprehensive understanding of the effects of synbiotics on liver function and overall metabolic health.

## Conclusion

Supplementation with multi-strain synbiotics, combined with lifestyle modifications, such as an individualized diet and physical activity program for 12 weeks, may serve as a promising adjunctive therapy to control disease progression in MASLD patients. This approach could lead to improvements in lipid profile (TC and LDL-C), FBS, liver function (ALT and AST), and liver inflammatory biomarkers (TNF-α and IL-6).

## Data Availability

The datasets used and/or analysed during the current study are available from the corresponding author on reasonable request.

## Abbreviations

ALP: Alkaline phosphatase
ALT: Alanine aminotransferase
AST: Aspartate aminotransferase
BMI: Body mass index
CONSORT: Consolidated standards of reporting trials
CRP: C-reactive protein
CVD: Cardiovascular disease
DBP: Diastolic blood pressure
FBS: Fasting blood sugar
FDA: Food and Drug Administration
FOS: Fructooligosaccharides
GGT: Gamma-glutamyl transpeptidase
Hb: Hemoglobin
HbA1C: Hemoglobin A1c
HCC: Hepatocellular carcinoma
HDL-C: High-density lipoprotein cholesterol
HOMA-IR: Homeostatic model assessment for insulin resistance
IBD: Inflammatory bowel disease
IBS: Irritable bowel syndrome
IL: Interleukin
INR: International normalized ratio
IRCT: Iranian Registry of Clinical Trials
LDL-C: Low-density lipoprotein cholesterol
MASLD: Metabolic dysfunction-associated steatotic liver disease
NAFLD: Nonalcoholic fatty liver disease
Plt: Platelet
PT: Prothrombin time
SBP: Systolic blood pressure
T2DM: Type 2 diabetes mellitus
TC: Total cholesterol
TG: Triglyceride
TNF-α: Tumor necrosis factor-alpha
WC: Waist circumference
WHtR: Waist-to-height ratio
